# Community environment and physical activity participation among rural older adults in China: evidence from the China longitudinal aging social survey

**DOI:** 10.3389/fpubh.2026.1753599

**Published:** 2026-01-27

**Authors:** Yong Zeng, Sheng Zhang, Daiming Li, Aidi Liao

**Affiliations:** 1 Physical Education Department, Xiangtan University, Xiangtan, China; 2 Department of Political Science and Public Administration, School of Public Administration, Xiangtan University, Xiangtan, Hunan, China

**Keywords:** China, class, community environment, physical activity, rural older adults

## Abstract

**Background:**

Physical activity is essential for improving population health, and participation levels are a key indicator of national social sports development. However, extant literature has largely focused on individual characteristics, with limited attention to the influence of the community environment. This study investigates how the community environment shapes physical activity among rural older adults in China and explores the mechanisms underlying these effects, providing evidence to support strategies aimed at promoting activity participation among rural older adults.

**Methods:**

Data from 5,668 respondents were obtained from the 2023 Chinese Longitudinal Ageing Social Survey. Binary logistic regression was applied to evaluate the influence of key community environmental attributes, including the availability of fitness facilities, the presence of outdoor activity spaces, and the provision of activity guidance, on physical activity participation among rural older adults in China after controlling for socio-demographic variables.

**Results:**

Among rural older adults, age, gender, educational level, marital status, and total personal income were significantly associated with physical activity participation, whereas living arrangement and self-rated health were not statistically significant. Access to fitness facilities was associated with a higher likelihood of engaging in physical activity, outdoor activity spaces showed a modest positive association, and activity guidance exhibited the strongest association.

**Conclusion:**

The community environment, including access to fitness facilities, outdoor activity spaces, and activity guidance, is associated with promoting physical activity among rural older adults. Strengthening the development of fundamental activity facilities in rural communities and accelerating the establishment of a community-based physical activity instructor system are recommended to advance the implementation of the national fitness strategy.

## 1 Introduction

Physical activity (PA) has been shown to provide a wide range of health benefits. It can help prevent chronic diseases such as cardiovascular disease and obesity, improve functional ability (1–5), and reduce the risk of mortality (6–9), while physical inactivity has been identified as the fourth leading risk factor for death (10). Regular physical activity is even more important for older adults. The global population of older people is increasing and is projected to reach 1.4 billion by 2030 (11). In most countries, however, physical activity decreases with age (12), with around 67% of people aged 65–74 and 75% of people over 74 not meeting recommendations (13). According to China’s National Fitness Activity Survey Bulletin, only 26.1% of the older adult population participates in regular physical activity in China (14). Physical activity is influenced by multiple factors. According to Giddens’ structuration theory, structure consists of rules and resources that actors can draw upon in different contexts, and structure is both constraining and enabling (15). Therefore, the physical activity of residents is shaped by both individual characteristics (actors) and the social environment (structure).

Research on the correlates of physical activity among older adults has mainly concentrated on two domains: individual characteristics and external environmental factors. In terms of individual characteristics, previous studies have consistently highlighted age, gender, marital status, education, income, and health awareness are important factors influencing physical activity (16–19). Many studies report that men are more likely than women to participate in physical activity, which is often explained by the dual roles women take on at home and in the workplace, reducing their available time and opportunities for physical activity (20). Age is another widely recognized determinant of physical activity. Research consistently shows that participation declines as individuals grow older, mainly because physical function gradually decreases (21). Evidence on the relationship between marital status and physical activity is inconsistent. Some studies report higher total activity levels among single adults (22, 23), while others find that married individuals, especially men, are more active. These differences often arise from variations in activity intensity rather than duration (24, 25). Older adults with higher education are more aware of the benefits of physical activity for health, functional ability, and quality of life, which increases their motivation to participate (26, 27). Income is also relevant, but its role is more complex. Some studies suggest that higher income is linked to greater participation in physical activity (28), while others, including the study by Lera-López and Rapún-Gárate, report no significant association (29). Additional evidence indicates that physical activity may even decrease at higher income levels, partly because better-paid jobs often involve longer working hours and less leisure time (30).

External environmental factors constitute important conditions that either constrain or promote physical activity, as most physical activity occurs within daily living spaces such as the home, community, and neighborhood (31). From the perspective of social support theory, both instrumental support and emotional support are important sources of motivation for individuals to maintain active and healthy leisure behaviors (32, 33). The community environment provides the material resources and contextual conditions necessary for physical activity (34–36). Previous studies have identified direct associations between physical environments and physical activity (37–39). Features such as adequate sidewalks, accessible shops, low traffic barriers, the availability and quality of sports facilities, and a higher density of physically active populations have all been shown to be positively related to physical activity (40–46). However, improving the physical environment alone is not sufficient to promote healthy behaviors (47–49). It is important to consider the interaction between physical environments (e.g., infrastructure for physical activity) and non-physical environments (e.g., financial costs or peer support) (50). A substantial body of evidence indicates that positive neighborhood relations act as an important facilitator of physical activity in community settings. Higher levels of community cohesion can create more opportunities for participation in physical activity (51), and a stronger sense of belonging further increases the likelihood that individuals will take advantage of these opportunities. These factors collectively contribute to greater engagement in physical activity (52–58). In contrast, weak neighborhood relations and low levels of community cohesion reduce the likelihood of participating in physical activity (59).

While previous studies drawing on individual and social environmental perspectives have provided valuable theoretical and empirical insights into physical activity among the rural older adults, several important gaps remain. First, much of the existing research has focused on factors that individuals can directly control or modify, such as marital status, income, education, and health awareness. Macro-level structural conditions, including the allocation of community resources, have received relatively limited attention. Even when community characteristics are included, they are typically measured as a single composite indicator (60), with limited attention to which specific components influence physical activity or how they exert their effects. Second, many studies have concentrated on adolescents, and empirical evidence on the relationship between the physical activity environment and physical activity among the rural older adults remains insufficient (61, 62). To address these gaps, this study uses data from the Chinese Longitudinal Ageing Social Survey to examine whether the community physical activity environment promotes physical activity among the rural older adults and to further explore the pathways through which these effects may occur.

## 2 Methods

### 2.1 Data and sample

Data were obtained from the Chinese Longitudinal Ageing Social Survey (CLASS),^1^ conducted by the National Survey Research Center at Renmin University of China. The CLASS was stratified, multi-stage, probabilistic sampling survey, which coved 28 provincial areas in China. Details of the design and conduct of the study have been described elsewhere (63). A total of 11,670 older adults aged 60 or above were interviewed by face to face. Following the study definition, rural older adults were identified as individuals aged 60 years and above with an agricultural *hukou* status. After excluding respondents with non-agricultural *hukou* and missing values, the final analytical sample comprised 5,668 rural older adults participants.

### 2.2 Measures and variables

#### 2.2.1 Physical activity

According to the conceptual definition of physical activity (64), engaging in physical activity less than once per month is considered occasional rather than regular (65, 66). Therefore, such behavior was classified as non-participation. Physical activity participation was measured with the survey question, “How often do you engage in physical activity?” Respondents who reported engaging in physical activity less than once per month were coded as non-participants (coded as 0), whereas those who engaged in physical activity at least once per month were coded as participants (coded as 1).

#### 2.2.2 Access to fitness facilities, outdoor activity spaces, and activity guidance

Access to the community environment for physical activity was measured using three survey-based variables. First, access to fitness facilities was assessed by the question, “Does your community have fitness venues or facilities?” with responses coded as 1 for “yes” and 0 for “no.” Second, access to outdoor activity spaces was measured by asking, “Does your community have outdoor activity spaces?” with the same coding. Third, activity guidance was assessed with the question, “Is there anyone who provides guidance when you engage in physical activity?” Respondents who answered “yes” were coded as 1, and those who answered “no” were coded as 0.

#### 2.2.3 Socio-demographic variables

Empirical evidence has shown that age, gender, educational level, marital status, living arrangement, total personal income, and self-rated health may be associated with physical activity participation among older adults (21, 67). Therefore, the following socio-demographic variables were controlled in this study: age (continuous variable), gender (1 = male, 0 = female), educational level (continuous variable), marital status (1 = with spouse, 0 = without spouse), living arrangement (1 = living with someone, 0 = alone), total personal income (RMB, log-transformed), and self-rated health (from 1 = very poor to 5 = excellent).

## 3 Results

### 3.1 Descriptive results

All statistical analyses were conducted using Stata 18.0. Table 1 reports the descriptive characteristics of the 5,668 rural older adults included in the study. With respect to age composition, the largest proportion of respondents were aged 70–74 years (36.79%), followed by those aged 65–69 years (27.49%), 75–79 years (15.46%), 80 years and above (10.71%), and 60–64 years (9.56%). The gender distribution was relatively balanced, with 53.44% male and 46.56% female. The mean years of education were 4.38 (SD = 3.15), indicating generally low educational level among the rural older adults. Most respondents reported having a spouse (79.75%), and the majority lived with others (89.52%). The mean log-transformed total personal income was 9.20 (SD = 1.15). Regarding self-rated health, 43.74% reported good health, followed by fair (39.43%), excellent (8.54%), poor (7.13%), and very poor (1.16%).

**Table 1:** Descriptive characteristics of the sample (*N* = 5,668).

Variables	n	%/ M (SD)
Age		
60–64	542	9.56
65–69	1,558	27.49
70–74	2085	36.79
75–79	876	15.46
80 and above	607	10.71
Gender		
Male	3,029	53.44
Female	2,639	46.56
Education level	5,668	4.38 (3.15)
Marital status		
With spouse	4,520	79.75
Without spouse	1,148	20.25
Living arrangement		
Alone	594	10.48
With someone	5,074	89.52
Total personal income	5,668	9.20 (1.15)
Self-rated health		
Very poor	66	1.16
Poor	404	7.13
Fair	2,235	39.43
Good	2,479	43.74
Excellent	484	8.54
Fitness facilities		
Available	386	6.81
Not available	5,282	93.19
Outdoor activity spaces		
Available	2,955	52.13
Not available	2,713	47.87
Activity guidance		
With guidance	1,046	18.45
Without guidance	4,622	81.55
Physical activity participation		
Yes	2,468	43.54
No	3,200	56.46

M, mean; SD, standard deviation.

With respect to community environmental factors, 52.13% of the rural older adults reported access to outdoor activity spaces. In contrast, access to community fitness facilities was extremely limited, with only 6.81% reporting availability. Furthermore, 81.55% of respondents lacked access to activity guidance, suggesting substantial gaps in community-based support for promoting physical activity.

Regarding physical activity participation, 43.54% of the rural older adults engaged in physical activity at least once per month, whereas 56.46% reported no participation.

### 3.2 Regression results

We employed binary logistic regression to examine how the community environment influences physical activity among the rural older adults. The analysis followed a stepwise approach, progressively incorporating sociodemographic variables and community environmental variables into four models (Table 2).

**Table 2:** Results of community environment on physical activity among rural older adults.

Variables	Model 1 b (SE)	Model 2 b (SE)	Model 3 b (SE)	Model 4 b (SE)
Age	−0.028***	−0.029***	−0.028***	−0.019***
	(0.005)	(0.005)	(0.005)	(0.006)
Gender	0.036	0.043	0.045	0.129*
	(0.055)	(0.056)	(0.056)	(0.061)
Education level	0.024**	0.021*	0.020*	0.021*
	(0.009)	(0.009)	(0.009)	(0.010)
Marital status	0.223*	0.230*	0.234**	0.260**
	(0.090)	(0.090)	(0.090)	(0.099)
Living arrangement	0.092	0.096	0.100	0.185
	(0.112)	(0.113)	(0.113)	(0.122)
Total personal income	−0.056*	−0.060*	−0.053*	−0.158***
	(0.024)	(0.024)	(0.024)	(0.027)
Self-rated health	0.007	0.003	0.002	0.053
	(0.035)	(0.035)	(0.035)	(0.038)
Fitness facilities		0.502***	0.490***	0.392***
		(0.107)	(0.107)	(0.118)
Outdoor activity spaces			0.111*	0.037
			(0.055)	(0.060)
Activity guidance				2.459***
				(0.095)
Constant	1.938***	2.002***	1.855***	1.551**
	(0.500)	(0.501)	(0.507)	(0.547)
N	5,668	5,668	5,668	5,668
pseudo R2	0.00977	0.0126	0.0132	0.135

*p < 0.1, **p < 0.05, ***p < 0.01.

Model 1 included only sociodemographic characteristics. Age was negatively associated with physical activity (*β* = −0.028, *p* < 0.01), indicating that older individuals were less likely to participate. Education level was positively associated with physical activity (*β* = 0.024, *p* < 0.05), and marital status also showed a positive association (*β* = 0.223, *p* < 0.1). Total personal income demonstrated a negative association (*β* = −0.056, *p* < 0.1). Gender, living arrangement, and self-rated health were not significantly associated with physical activity.

Model 2 added access to fitness facilities. Fitness facilities showed a strong positive association with physical activity (*β* = 0.502, *p* < 0.01). The associations of age, education level, marital status, and income remained consistent with Model 1, and gender, living arrangement, and self-rated health continued to be non-significant.

Model 3 added access to outdoor activity spaces. Outdoor activity spaces were positively associated with physical activity (*β* = 0.111, *p* < 0.1), although the effect was smaller than that of fitness facilities. The associations of other variables were generally consistent with those observed in Model 2.

Model 4 further incorporated activity guidance. Activity guidance showed a very strong and significant association with physical activity (*β* = 2.459, *p* < 0.01), indicating an important role of guidance in supporting participation among the rural older adults. After activity guidance was included, the effect of outdoor activity spaces became non-significant, whereas gender became significant (*β* = 0.129, *p* < 0.1), suggesting that men were more likely to engage in physical activity when community-level support was considered. Education level, marital status, and income remained significant, while living arrangement and self-rated health continued to show no significant associations.

## 4 Robustness test

To assess the robustness of the main results, we re-estimated the model using a probit specification. As shown in Table 3, the probit estimates were consistent with those from the binary logit model. The direction and statistical significance of all key variables remained stable across the two specifications. In particular, age, gender, education level, marital status, total personal income, fitness facilities, and activity guidance continued to show significant associations with physical activity. Although the coefficient magnitudes differed between the binary logit and probit models, these differences reflect the scaling of the two estimation methods and do not affect the substantive conclusions. Overall, the findings indicate that the main results are robust to the choice between binary logit and probit models.

**Table 3:** Results of robustness test.

Variables	Model 1	Model 2
Age	−0.019***	−0.012***
	(0.006)	(0.003)
Gender	0.129*	0.078*
	(0.061)	(0.037)
Education level	0.021*	0.013*
	(0.010)	(0.006)
Marital status	0.260**	0.155**
	(0.099)	(0.059)
Living arrangement	0.185	0.111
	(0.122)	(0.074)
Total personal income	−0.158***	−0.094***
	(0.027)	(0.016)
Self-rated health	0.053	0.030
	(0.038)	(0.023)
Fitness facilities	0.392***	0.223**
	(0.118)	(0.071)
Outdoor activity spaces	0.037	0.020
	(0.060)	(0.036)
Activity guidance	2.459***	1.475***
	(0.095)	(0.052)
Constant	1.551**	0.920**
	(0.547)	(0.330)
N	5,668	5,668
pseudo R2	0.135	0.135

*p < 0.1, **p < 0.05, ***p < 0.01.

## 5 Discussion

The significant negative association between age and physical activity is consistent with previous research showing that participation declines as individuals grow older (68). For instance, research shows many older adults are physically inactive, including 42% in Poland, less than a quarter in Brazil, and nearly half in rural Thailand (69, 70). This pattern may reflect a combination of physical, psychological, social, and economic factors. Physically, declining health and functional limitations reduce the capacity to engage in activity. Psychologically, motivation and attitudes toward physical activity may shift with age. Social norms and stereotypes about ageing can also shape expectations regarding appropriate activity levels. Economically, reduced disposable financial resources in later life may further constrain opportunities for regular physical activity.

In Models 1–3, gender was not significantly associated with physical activity, suggesting that the difference between men and women may be obscured when only sociodemographic characteristics and basic community resources are controlled. After incorporating activity guidance in Model 4, gender became significant, indicating that men benefit more from structured or professional exercise guidance and show a greater increase in physical activity participation than women. Thus, activity guidance plays a moderating role that amplifies the gender difference and uncovers the previously hidden effect of gender on physical activity.

This study also finds that rural older adults with higher levels of education are more likely to engage in physical activity, which is consistent with previous evidence linking educational level to healthier behavioral choices (71). One possible explanation is that higher education improves their awareness of the health and functional benefits of physical activity, thereby strengthening motivation to exercise. Moreover, rural older adults with higher education tend to possess greater social and economic resources, such as more stable financial conditions and better living environments, which provide more favorable opportunities and support for participating in physical activity.

Rural older adults individuals with a spouse are more likely to engage in physical activity. From the perspective of social support theory, spouses provide emotional encouragement, practical assistance, and routine reminders that reinforce healthy behaviors (72). As physical functioning declines with age, dependence on companionship and support becomes increasingly important, particularly among those aged 70 years and above (73, 74). Previous evidence further indicates that couple-based interventions, which involve both members of a married pair, can be especially effective in increasing physical activity participation and promoting long-term adherence among older adults (75).

In this study, living arrangements were not significantly related to physical activity, which contrasts with several previous findings (76–79). A possible reason is that in rural China, co-residence is shaped more by caregiving responsibilities rather than emotional support. Rural older adults who live with their children are often responsible for grandchild care and household tasks, which reduces the time and autonomy available for engaging in physical activity. As a result, living with others may not provide the encouragement or companionship that supports physical activity. This explanation is also supported by evidence from some previous studies (80, 81).

This study reveals a significant negative association between total personal income and participation in physical activity among the rural older adults. Although this finding may appear counterintuitive, it is consistent with the income-leisure trade-off theory. Descriptive statistics show that 84.81% of respondents were engaged in agriculture, animal husbandry, or fishery, and 59.47% reported feeling “quite tired” or “very fatigued” due to work-related physical and mental demands. These patterns suggest that the rural older adults are primarily involved in high-intensity manual labor. Under such occupational conditions, higher income is typically associated with longer working hours and greater labor intensity. These income-generating activities not only reduce available leisure time but also contribute to persistent physical fatigue (82). From a rational choice perspective, minimizing non-productive physical activity becomes a utility-maximizing strategy when energy is limited, reflecting a behavioral tendency to prioritize rest and recovery over additional exercise.

The findings show that in rural China, self-rated health does not significantly promote participation in physical activity. This may be because the exercise behavior of the rural older adults is largely shaped by external factors such as lifestyle patterns, labor demands, cultural norms, economic conditions, and the availability of activity spaces. As a result, better self-rated health does not necessarily translate into a greater ability or willingness to engage in physical activity.

This study also found that fitness facilities are significantly associated with the physical activity behaviors of the rural older adults. Two reasons may explain this association. First, accessible and age-friendly sports facilities can reduce the barriers that older adults individuals face when engaging in exercise as their physical function declines, providing safer and more convenient opportunities for activity (83, 84). Second, due to the limited availability of public exercise resources in rural areas, the accessibility and quality of sports facilities become particularly important for encouraging physical activity among the rural older adults. Community sports resources can provide platforms for social interaction, facilitating the formation of activity partners or teams (85, 86). In other words, the social environment enhances interpersonal connections within the community, which further promotes participation in community activities (87).

Outdoor activity spaces are associated with physical activity among the rural older adults to some extent, although the association is relatively limited in rural settings. One explanation is that rural older adults typically have access to spacious private courtyards and abundant natural environments, which provide greater privacy, easier accessibility, and higher convenience. These informal spaces substantially substitute for formal outdoor activity spaces, reducing their actual use. In addition, the quality of outdoor activity space design, including plant density, structural enclosure, and functional mixing (88), is often more important than the mere presence of such spaces. Previous studies have reported that when physical environments lack features that are necessary or attractive for specific activities, individuals may lose opportunities to engage in physical activity (89). Other studies have further suggested that although access to supportive environments is a necessary condition, it is insufficient for adults to achieve recommended levels of physical activity (90). Therefore, providing outdoor activity spaces alone is unlikely to substantially increase physical activity among the rural older adults. This interpretation is consistent with our findings, which show only a marginally significant association in Model 3 and a non-significant effect once activity guidance is included in Model 4.

Research on physical activity guidance for rural older adults populations consistently demonstrates that supervised exercise programs are safe and effective in enhancing physical function (91, 92). Such improvement is essential for maintaining independence and quality of life, with particularly significant benefits observed in women (92). In rural China, these findings can be interpreted through two key mechanisms. First, activity guidance can help reduce risks related to physical activity for rural older adults, who generally have lower education levels and limited knowledge in this area, which in turn can increase their participation rates. Second, organized group activities such as square dancing and Tai Chi create stable social platforms that help alleviate the widespread empty-nest phenomenon by fulfilling social needs and enhancing exercise motivation. Third, we note that the cross-sectional design of this study may involve selection bias, as individuals who are already physically active, motivated, or socially connected may be more likely to seek or engage in activity guidance. Taken together, this evidence underscores the importance of accelerating the development of community-based fitness instructor systems to advance national fitness initiatives.

Several limitations should be acknowledged when interpreting the findings of this study. First, the analysis was based on cross-sectional data, which limits the ability to capture long-term changes in community environments and their cumulative effects on physical activity among the rural older adults. Second, the measurement of community environment was restricted by the indicators available in the dataset. Although access to fitness facilities, outdoor activity spaces, and activity guidance reflects certain aspects of community resources, these indicators cover only a small portion of the overall community environment. Key components such as environmental safety, transportation accessibility, walkability, and community participation were not included, which may weaken the explanatory power of the study. Third, regular physical exercise is typically defined as engaging in activities at least once a week, lasting at least 30 min, and of moderate intensity. However, in the current study, participants were defined as those who engaged in physical activity at least once per month, which represents a much lower threshold compared to commonly recommended guidelines. Fourth, future research should incorporate additional determinants of physical activity that have been widely validated in previous studies, including health awareness, medical insurance coverage, and chronic disease management.

Despite these limitations, this study is, to our knowledge, the first to use nationally representative data to examine how community environments affect physical activity among rural older adults in China. The findings broaden the literature by highlighting community-level influences and offer useful evidence for policies that support healthy aging in rural areas.

## 6 Conclusion

This study demonstrates that both individual and community environment factors are associated with physical activity among the rural older adults. At the individual level, age, gender, education level, marital status, and total personal income showed significant associations with physical activity, whereas living arrangement and self-rated health showed no clear associations. At the community level, the environment also played an important role, with access to fitness facilities, outdoor activity spaces, and activity guidance contributing to higher levels of participation. Based on these findings, strengthening the development of activity facilities in rural communities and accelerating the establishment of a community-based physical activity instructor system are recommended to promote physical activity among the rural older adults and to support the implementation of the national fitness strategy.

## Data Availability

Publicly available datasets were analyzed in this study. This data can be found at: The datasets presented in this study can be found in online repositories. The name of the repository and accession information can be found at: the data of the study are publicly available and can be accessed via the China Longitudinal Aging Social Survey (CLASS) website: https://class.ruc.edu.cn.

## Footnotes

1
http://class.ruc.edu.cn
